# Isotope effect in superconducting n-doped SrTiO_3_

**DOI:** 10.1038/srep37582

**Published:** 2016-11-28

**Authors:** A. Stucky, G. W. Scheerer, Z. Ren, D. Jaccard, J.-M. Poumirol, C. Barreteau, E. Giannini, D. van der Marel

**Affiliations:** 1 Department of Quantum Matter Physics, Université de Genève, CH-1211 Genève 4, Switzerland

## Abstract

We report the influence on the superconducting critical temperature *T*_*c*_ in doped SrTiO_3_ of the substitution of the natural ^16^O atoms by the heavier isotope ^18^O. We observe that for a wide range of doping this substitution causes a strong (~50%) enhancement of *T*_*c*_. Also the magnetic critical field *H*_*c*2_ is increased by a factor ~2. Such a strong impact on *T*_*c*_ and *H*_*c*2_, with a sign opposite to conventional superconductors, is unprecedented. The observed effect could be the consequence of strong coupling of the doped electrons to lattice vibrations (phonons), a notion which finds support in numerous optical and photo-emission studies. The unusually large size of the observed isotope effect supports a recent model for superconductivity in these materials based on strong coupling to the ferroelectric soft modes of SrTiO_3_.

SrTiO_3_ is a para-electric insulator which becomes ferroelectric when 35% or more of the oxygen is substituted with the isotope ^18^O^1^,^2^,^3^. Due to electron-phonon coupling doped charge carriers form a polaronic liquid at small concentration^4^,^5^,^6^,^7^,^8^,^9^,^10^,^11^,^12^,^13^ and the material becomes superconducting with a doping dependent critical temperature (*T*_*c*_) below ~1 Kelvin both for bulk^14^,^15^,^16^,^17^,^18^,^19^ and interfaces^20^,^21^. Several aspects of this material are not usually encountered in conventional superconducting metals. These include a multi-valley bandstructure^22^ (however presently known not to apply to SrTiO_3_), the structural transition at around 100 K^23^, and the low density of charge carriers^4^,^24^,^25^,^26^,^27^. More recently a connection between the near ferroelectric instability and the superconductivity of SrTiO_3_ has been conjectured^3^,^28^.

Here we report on the superconducting properties of doped SrTiO_3−*y*_ with partial ^18^O/^16^O isotope substitution, and observe that substituting 35% of the heavier ^18^O for ^16^O increases *T*_*c*_ by a factor of approximately 1.5. The sign of the observed isotope effect is opposite to one in conventional superconductors and the magnitude much stronger. The unusual size and sign of the isotope effect may be caused by the near ferroelectric instability, the polaronic nature of the charge carriers, or a combination of those.

Undoped SrTiO_3_ crystals were annealed under pure ^18^O_2_ atmosphere. During this procedure, part of the ^16^O atoms in the structure is substituted by ^18^O atoms. The isotope substitution ratio has been quantified by three independent and complementary experimental techniques. We estimate the amount of isotope substitution to be 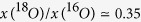 (see *Methods*). N-type doped samples were prepared by subjecting the samples to a reduction process. Three samples were made with charge carrier density of n = 0.004 *nm*^−3^, n = 0.02 *nm*^−3^ and n = 0.07 *nm*^−3^ representative of a large portion of the phase diagram. At each doping level of SrTi^18^O_3−*y*_, one parent SrTi^16^O_3−*y*_ sample was annealed under the same conditions and at the same time, thus providing a reference for the isotope effect on the superconducting properties. Details of preparation and characterization can be found in *Methods*. The samples were cooled down to 25 mK in a ^3^He-dilution refrigerator. A current between 10 and 50 mA (corresponding to a density between 1.6 and 8 A/cm^2^) was flowing in the sample. The longitudinal voltage was acquired to probe the intrinsic resistivity; the resulting *ρ(T*) curves are reported in Fig. 1(a–c). The “onset” transition temperatures are extracted from this data as the crossing point between the linear extrapolation of the normal state resistivity above *T*_*c*_ and the linear extrapolation of the resistivity drop at the transition. The error bar is estimated as the temperature range over which the derivative *dρ(T*)/*dT* changes. Figure 1(e) shows the extracted transition temperatures as a function of the charge carrier density for all samples. The values of *T*_*c*_ indicate a systematic increase of the superconducting transition temperature with the presence of the heavier isotope. Figure 1(d) shows the magnetic AC-susceptibility for the two highly doped samples. This was measured in a two-coil set-up whose pick-up coil voltage change was amplified by a standard lock-in (feeding current 0.05 mA at a frequency of 977 Hz, providing a magnetic field of 0.014 mT)^29^. The transition temperatures, obtained from transport (panel (c)) and AC-susceptibility measurements (panel (d)), well agree with each other.

The BCS weak coupling limit gives an isotope coefficient *α* = −*d*(ln *T*_*c*_)/*d*(ln *M*) = 0.5 (where *M* is the oxygen isotope mass), corresponding to a *T*_*c*_ shift of −5% as opposed to approximately +50% in the data presented here. The first main observation is therefore that, contrary to most superconducting materials, the isotope coefficient in SrTiO_3_ is negative. In some other rare cases *α* < 0 has been observed: the pure uranium (*α* = −2.2)^30^, the high-*T*_*c*_ superconductor Bi_2_Sr_2_Ca_2_Cu_3_O_10_ (*α* = −0.1)^31^, and the metal hydride PdH(D)_*x*_^32^,^33^ (−0.3 < *α* < −0.1). Controversial sign changes of the isotope coefficient have been observed in (Ba,K)Fe_2_As_2_ (*α* = −0.2)^34^,^35^ (due to differences in the sample composition), and in pure lithium under high pressure (*α* changes with increasing pressure^36^). We will not dwell on the physical origins of the isotope effect in these cases, which are certainly different in the case of uranium, and possibly different in the other examples as well. Our second main observation is, that also the magnitude of the isotope effect is remarkable: an overall enhancement of *T*_*c*_ of a factor 1.5 is observed at all doping levels, which corresponds to a negative and large coefficient *α* ~ −10. This is possibly the strongest isotope enhancement of *T*_*c*_ observed in any material so far.

From the measurement of the electrical resistivity at the superconducting transition in magnetic field, we could estimate the upper critical field *H*_*c*2_(*T*) and the effect that the isotope substitution can have on it. These results are summarized in Fig. 2. For each sample we plot the onset of *ρ(T, H*), defined as the crossing point of the linear extrapolations of the normal state resistance and the slope of the resistance at the transition. The *H*_*c*2_(*T*) line of ^18^O-substituted SrTiO_3_ is far above that of the pristine sample, at each doping level. The isotope effect does not only enhance the critical temperature, but strengthens the superconductivity in a magnetic field as well, up to a maximum 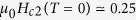 tesla at optimal doping. For all dopings the observed isotope effect on *H*_*c*2_ corresponds to *β* = −*d* ln *H*_*c*2_/*d* ln *M* ≈ −20. We notice that the samples with lower charge carrier density exhibit a higher critical field, despite of the lower critical temperature. This is not surprising and it is expected on the basis of the band structure reported in ref. 9 and the two-gap superconductivity reported for SrTi_1−*x*_Nb_*x*_O_3_^37^ (since gap anisotropy is in general affected by impurity scattering, this may *a priori* depend on the kind of doping, *e.g*. oxygen depletion, Nb substitution or La substitution). As a matter of fact, the different doping levels shown here correspond to the occupation of different bands^9^, which contribute differently to the superconducting pairing. The temperature dependence of the critical field does not change even when the critical temperature and field are enhanced by the isotope substitution.

We measured the resistivity under a magnetic field in both orientations perpendicular to the current (with the current always in the same direction in the *ab*-plane of the crystal). Both measurements provide the same result, thus ruling out any possible surface effect on the superconducting behavior. The lowermost panel of Fig. 2 shows the two identical *H*_*c*2_(*T*) transition lines measured in the two different field orientations.

There are three infrared active modes in SrTiO_3_, having transverse frequencies 67, 21 and 2 meV and Fröhlich coupling constants *α*_*ep*_ = 1.6, 0.45 and 0.02, respectively (see Table 1 of ref. 38). The (Fröhlich type) electron-phonon coupling in SrTiO_3_ is dominated by coupling to the optical phonons at 67 and 21 meV^8^,^38^; these phonons are to a large extend responsible for the twofold mass-enhancement of the charge carriers observed with optics^7^,^38^. Note that the coupling constant of the soft ferroelectric mode at 2 meV can not be correctly estimated from the Fröhlich mode; it is certainly larger, among other things due to the strong anharmonicity of this mode^28^,^39^. The doping dependence of the effective mass in units of the bare band-mass is described by the phenomenological expression^9^ m*/*m* = 2.0 + 1.2 exp(−*n/n*_0_), where *n*_0_ = 0.084 nm^−3^ is an empirical factor. For all doping concentrations where superconductivity is observed the quasiparticles are slow compared to the vibrational degrees of freedom^9^, which corresponds to the anti-adiabatic limit. The quasiparticles in this limit are commonly referred to as “polarons”. In case the interactions are not too strong the polarons form a Fermi-liquid. Alexandrov^40^ has pointed out in this context that the critical temperature of the superconducting transition is determined by a BCS-like formula, but with the renormalized density of states replacing the bare one. In conventional models of electron-phonon coupling the effective coupling constant describing the pairing interaction, *λ*, is invariant under isotope substitution. (We use a single parameter *λ* to describe the combined phonon mediated and Coulomb interaction). However, this does not take into account aforementioned band-renormalization, due to which it should be replaced with the effective parameter


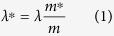


The corresponding expressions for the isotope coefficient are detailed in Eqs 4 and 9 of the Methods section. The parameters in Table 1 imply that *α* ~ −1.7 ± 0.3, which is larger than the BCS result and of opposite sign. For the isotope coefficient of the upper critical field the model gives *β* = −*d*(ln *H*_*c*2_)/*d*(ln *M*) ~ −3.9 ± 0.5.

Hence the unusual isotope effect that we observe in the experiments is in principle not unexpected given the polaronic nature of the charge carriers. Note that *T*_*c*_ is a very sensitive function of λ* due to the fact that in STO this parameter (and consequently also *T*_*c*_) is very *small*. The situation is quite different in this respect from that in cuprate high temperature superconductors, for which the ^18^O isotope substitution causes *T*_*c*_ to become smaller^41^,^42^. The fact that in SrTiO_3_ the experimental isotope coefficients *α* ~ −10 and *β* ~ −20 are still considerably larger than expected from aforementioned band-renormalization, calls for deeper theoretical analysis of the electronic structure and the effective polaron-polaron interactions in these materials. A possible clue comes from the recent theoretical study by Edge *et al*.^28^, whom postulated that pairing of electrons in SrTiO_3_ is driven by the ferroelectric soft mode fluctuations in the proximity of a quantum critical phase transition. Following Kedem *et al*.^39^ the coupling parameter close to the ferroelectric quantum critical point is


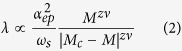


where *α*_*ep*_ is the electron-phonon coupling constant, *ω*_*s*_ a vibrational energy scale, *M*_*c*_ the mass at the critical point and *zν* the critical exponent of the system having the mean-field value *zν* = 0.5. For harmonic modes 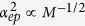, which leads to a mass independent 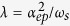 since also *ω*_*s*_ ∝ *M*^−1/2^. Following the description of Edge *et al*. the effect of doping is to increase *M*_*c*_, which for the relevant doping range (>10^19^ cm^−3^) falls above the mass of ^18^*O*. Consequently at these doping levels there is no ferroelectric instability (corresponding to a zero in the denominator) for any (partial or complete) ^18^*O* isotope substitution. Yet, according to Eq. (2), even far from the ferroelectric instability *λ* is a sensitive function of the isotope mass. Based on these considerations Edge *et al*. predicted a 30% increase of *T*_*c*_ for 35% isotope substituted samples. Note that the renormalization of *λ* due to polaronic band narrowing occurs regardless of the pairing mechanism, hence Eqs (1) and (2) need to be combined. Taken together these two effects do indeed account -with a large margin- for the observed value, *α* ~ −10, of the isotope coefficient.

We have studied the critical temperature *T*_*c*_ and the upper critical field *H*_*c*2_ of partially (35%) ^18^O isotope substituted doped SrTiO_3_. We observe a strong (factor ~ 1.5) enhancement of *T*_*c*_ and (factor ~ 2) of *H*_*c*2_. Both the sign and the size of these two effects are unusual, and indicate that the superconductivity is not of the conventional BCS variety. The effect may be the consequence of a combination of two effects: polaronic band-narrowing and coupling to soft phonons responsible for the ferroelectric instability in these materials.

## Methods

### Sample processing

For the present study, we chose SrTiO_3_ samples from various sources and different morphologies: commercial crystals of pure SrTiO_3_, commonly purchased as substrates for thin film deposition by MTI Corp.; superconducting crystals of Nb-doped SrTiO_3_, with a Nb:Ti nominal ratio ranging from 0.001 to 0.05, from CrysTech; home-processed ceramic samples of SrTiO_3_ obtained from binary reaction between the binary oxide components. The isotope substitution of ^18^O for ^16^O in crystalline samples was achieved following a procedure similar to that reported by Itoh *et al*.^2^. We carried out a three step cycle where in each step the crystals were re-annealed in a new sealed quartz reactor filled with pure ^18^O_2_. Based on the amount of oxygen in the sample (between 5 · 10^−5^ and 6.5 · 10^−5^ *mol*), and the volume of the quartz tubes (between 12 and 15 *cm*^3^), and assuming that equilibrium has been reached at the end of each of the 3 consecutive annealing steps, the expected value of ^18^O substitution would be between 99.4 and 99.9%. As a matter of fact, the procedure followed for substituting the isotope ^18^O for ^16^O was only partially effective and did not allow full isotope substitution. The actual amount of oxygen isotope substitution proved to be ~35%, as directly measured by three independent and complementary experimental techniques described in the following.

In the ceramic samples the isotope substitution was obtained by first oxidizing the pure metals Sr and Ti in pure ^18^O_2_ atmosphere, then reacting Sr^18^O and Ti^18^O_2_ to form SrTi^18^O_3_. Processing of ceramic samples was highly time-consuming and costly, and yielded only a small amount of SrTi^18^O_3_; however, this was a crucial reference for the calibration and optimization of the ^18^O substitution in the crystalline samples.

The isotope-substituted samples from the various sources were then subjected to reduction treatments (10^−7^ mbar at 1000°–1350 °C) in order to create oxygen vacancies and tune the doping of charge carriers. For each doping level of each ^18^O-substituted sample, a twin ^16^O-sample (either crystal or ceramic) was subjected to the same annealing conditions and reduction treatments, at the same time and in the same furnace. By virtue of this twin-treatment a complete range of SrTi^16^O_3_/SrTi^18^O_3_ twin samples was obtained. This is the optimal procedure to limit ambiguities due to sample processing when comparing the physical properties of the ^16^O- and ^18^O-samples. For the study of the superconducting behavior of SrTi^18^O_3_ through transport measurements in a dilution fridge, only oxygen-reduced single crystals of SrTiO_3_ were used. These samples, as well as their processing conditions are reported in Table 2.

### Determination of the isotope substitution level

The amount of ^18^O/^16^O substitution is commonly estimated from the mass uptake of the sample^2^. However, such an estimation is not free from uncertainties and ambiguities. We want to know how much ^18^O actually substitutes for ^16^O under the processing conditions used, and check wether the response of the material is that expected for the isotope-substituted ^18^O-oxide. We pursued this goal by three different techniques: mass spectroscopy, thermogravimetry and infrared optical spectroscopy. Mass spectrometry was performed on selected pieces of crystals and ceramic samples in the Stable Isotope Laboratory at the University of Lausanne.

These measurements confirmed that oxygen has been substituted throughout the bulk of the material, and indicated that the amount of ^18^O in the (ceramic and crystalline) samples is about 35%. The results of this analysis are summarized in Table 3. The increment of the substitution of ^18^O from 10% to ~35% between the first and the last step of the treatment is shown as well. Such an incomplete isotope substitution is unexpected, having followed a procedure previously reported to be effective for full substitution^2^. We will return to this point below.

In the thermogravimetric experiment, two crystals of SrTi^18^O_3_ and SrTi^16^O_3_ were simultaneously heated in the symmetric furnace of a Setaram TAG24 thermal analyzer under a flux of pure ^16^O_2_. The same thermal treatment as used for the substitution process was reproduced in the thermal analyzer. The mass loss of the former with respect to the latter corresponds to the loss of ^18^O, replaced back by the ^16^O during this treatment. The drift of the thermobalance as a function of time and temperature was measured from a similar thermogravimetric experiment in which two identical SrTi^16^O_3_ crystals were used as the sample and the reference, respectively. The mass loss associated to the loss of ^18^O is plotted in Fig. 3(a). The total amount of ^18^O substituted by ^16^O is found to be 35%, in good agreement with the mass spectrometry.

The isotope substitution is expected to affect the phonon modes, whose frequency shift can be measured by either infrared (IR) or Raman spectroscopy. If the reduced mass of an IR vibrational mode is that of the oxygen atoms, and assuming that everything else (lattice parameter, atomic positions, zero-point fluctuations) remains the same upon isotope substitution, the softening for a complete substitution of ^16^O by ^18^O would be given by 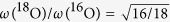, which corresponds to a red-shift of about 6%. The IR reflectivity of the pristine and substituted samples, measured in a Fourier transform infrared spectrometer, is shown in Fig. 3(a) over the energy range from 350 to 600 cm^−1^ (43.4 to 74.4 meV). The strong absorption at 480 cm^−1^ (59.5 meV) is associated to the TO4 phonon mode due to the axe zone-center displacement of the oxygen octahedra^43^ and is strongly dependent on the oxygen isotope mass. Figure 3(b) shows the softening of that mode in samples exposed to repeated thermal treatments under ^18^O atmosphere, thus proving that the isotope substitution has occurred. The measured shift is a factor three lower than the ~6% expected for a complete isotope substitution, confirming that the procedure followed for isotope substitution is effectively replacing only ~35% of ^16^O by ^18^O.

The red-shift of the phonon mode is found to be of the same magnitude in ceramic samples as in crystals, the former having being processed by oxidation of metal elements in pure ^18^O_2_, and confirms well the amount of substitution measured by the mass spectrometry and thermogravimetry. One would expect complete isotope substitution in ceramic samples. However, the reaction treatment to form SrTi^18^O_3_ from Sr^18^O and Ti^18^O_2_ is done in SiO_2_ reactors at high temperature, thus bringing it down to the same high temperature environment as used for crystalline samples. This leads us to suspect that part of the oxygen of the quartz tubes participates in the equilibrium. This could be a surface layer, or a small fraction of less stable bonded oxygen.

### Electron doping of SrTiO_3_

After the ^18^O-substitution, n-type charge carriers were introduced by creating oxygen vacancies through vacuum annealing. This is a widely used procedure, long known as being successful in tuning the carrier density and the conductivity of SrTiO_3−*y*_^17^,^44^. By optimizing the annealing temperature between 800 °C and 1400 °C and the annealing time between 20 and 36 h, we could span a wide range of charge doping, from 5 · 10^16^ to 2 · 10^20^ cm^−3^. This doping range corresponds to the underdoped side of the superconducting phase diagram up to the maximum critical temperature of ~300 mK at *n* = 10^20^ cm^−3^. The carrier density is obtained by measuring the Hall effect in a 5-probe configuration using a Quantum Design PPMS apparatus. The homogeneity of oxygen depletion is enhanced after long annealing time. According to the Hall characterization of a variety of samples subjected to different reducing treatments, we have selected, for this study, three pairs of crystalline samples (three with ^16^O ad three with ^18^O) with the same oxygen reductions, having been treated for 36 h at 1050°, 1200°, and 1350 °C. The charge carrier density (measured at 4 K) in oxygen reduced samples was 2 · 10^18^ cm^−3^, 4 · 10^19^ cm^−3^, and 10^20^ cm^−3^, for the three reduction temperatures, respectively.

**Figure 1:**
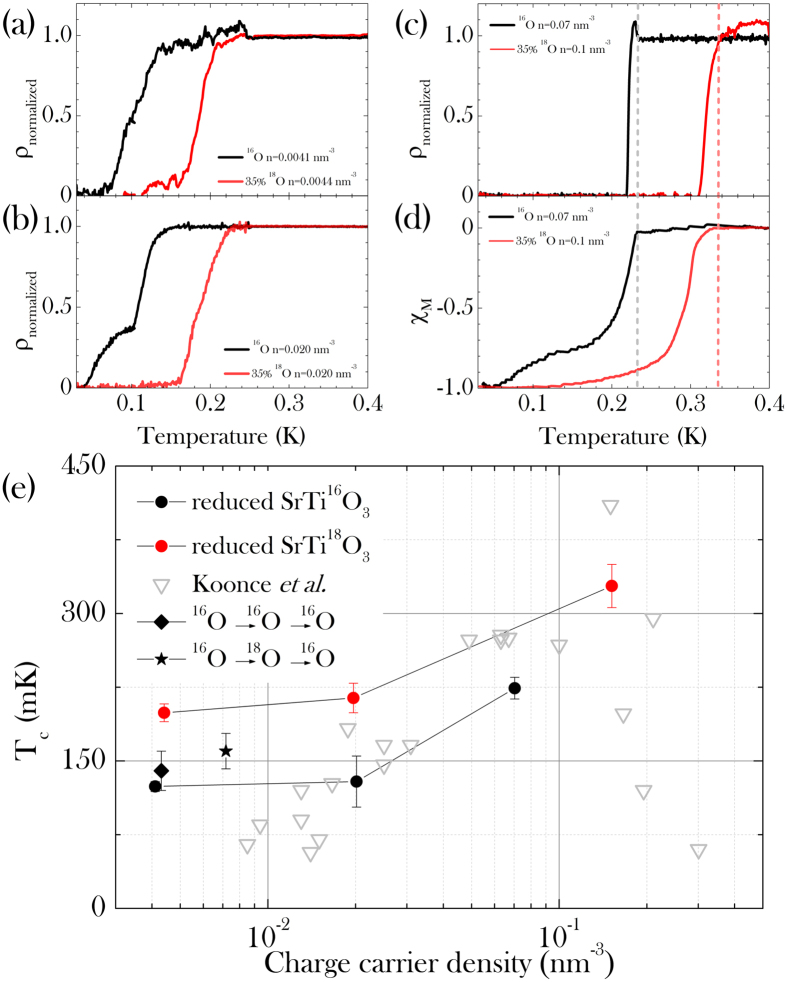
Normalized resistivity *vs*. temperature at the superconducting transition of three different doping levels: (**a**) *n* = 0.004 nm^−3^ (**b**) *n* = 0.02 nm^−3^ (**c**) *n* = 0.07 nm^−3^ (**d**) AC-Susceptibility showing the magnetic transition to the superconducting state of the same sample as in panel (**c**). (**e**) *T*_*c*_ *vs*. charge carrier density. Full symbols: experimental data of the present study for SrTi^18^O_3−*y*_ (red) and SrTi^16^O_3−*y*_ (black). Grey symbols: *T*_*c*_ values reproduced from ref. 17. Black diamond and star refer to samples in which ^16^O was back-substituted after isotope substitutions (see *Methods*).

**Figure 3:**
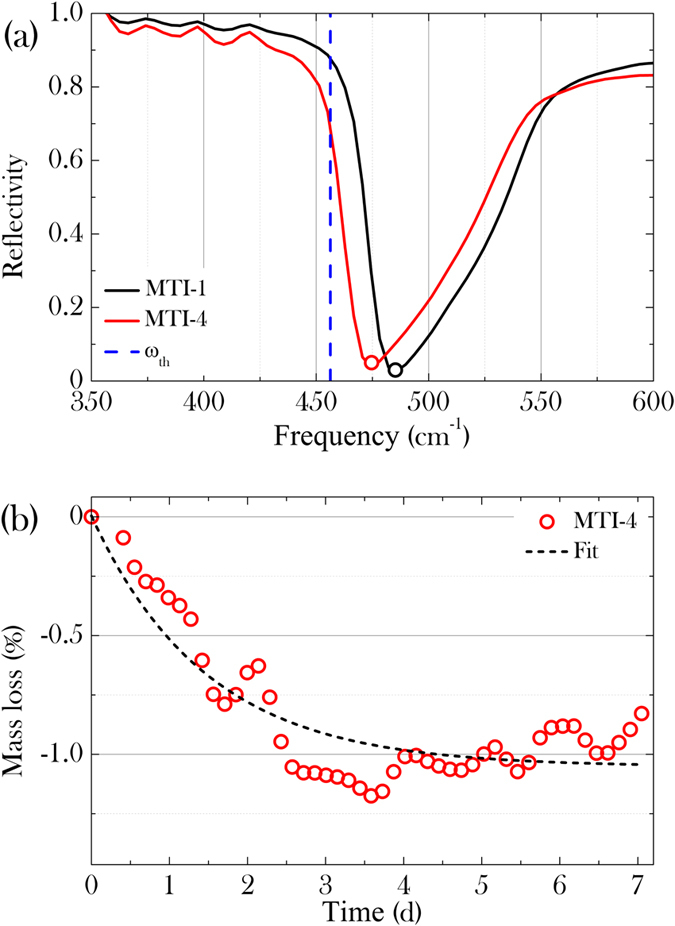
(**a**) Shift of the phonon mode in the IR spectrum, due to isotope substitution. The vertical dashed line correspond to the expected frequency shift in case of complete isotope substitution. (**b**) Mass loss due to reverse substitution of ^16^O for ^18^O as measured by thermogravimetry. The dashed line is a fit to an asymptotic exponential.

According to the literature^17^, these carrier densities are expected to correspond to superconducting critical temperatures of 100 mK, 200 mK and 300 mK, respectively (see Fig. 1(e)). The electrical resistivity as a function of temperature of various samples is displayed in Fig. 1(a–c), whereas the magnetic transition in the AC-susceptibility is shown in Fig. 1(d). The Nb-substituted crystals could not be used for the same study. The ^18^O-substitution treatment actually affect the Nb doping, thus modifying the charge carrier density in an uncontrolled way. Because of the impossibility to tune and control independently the isotope substitution and the charge carrier doping in Nb-doped SrTiO_3_, we selected only O-reduced crystals for this study.

### Back-substitution

**Figure 4:**
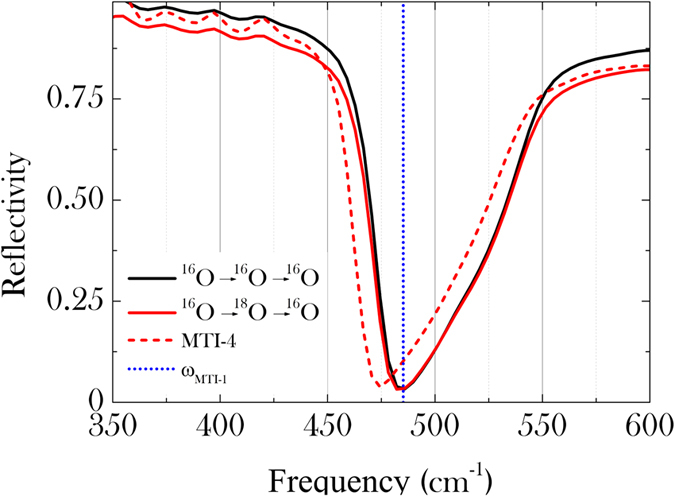
FIR spectroscopy of the two back-substituted samples (red and black lines). Red, respectively blue, dotted lines corresponding to the ^18^O substituted sample MTI-4, respectively to the phonon position of the pristine sample, are added as a reference for the phonon shift *T*_*c*_ *vs*. charge carrier density described in Fig. 1(e) with two supplementary points relative to the measurement of the samples back-substituted.

The two samples treated under flux of ^16^O_2_ for the characterization by thermogravimetry (MTI-1 and MTI-4) are used to study the physical properties after back substitution. Figure 4 shows the infrared reflectivity for the two back-substituted samples. No shift has been observed in the phonon frequency of back-substituted ^16^O and back-substituted ^18^O compared to the pristine sample. This confirms the fact that both samples are indeed completely ^16^O back-substituted. The same doping procedure by oxygen reduction as the one for samples MTI-1 and MTI-4 (see Table 2) has been applied to the back-substituted samples. Electric transport measurements down to 30 mK and Hall characterization were performed in parallel on the two samples using the same procedure as presented in Fig. 1(a–c). The results, obtained following the same way to extract the critical temperatures and to calculate the charge carrier densities, are compared to the substituted samples in the Fig. 1(e). The general rise of *T*_*c*_ of the back-substituted samples indicates that repeated annealing improves the quality of the materials. Despite performing the annealing of the two back-substituted samples simultaneously in the same furnace, their electron concentration is different. This is explained by the fact that, due to the unavoidable step of balance calibration in the thermo-gravimetry set-up, the back-substitution process has been repeated twice for the ^16^O sample. Thereby, the two back-substituted samples didn’t have the same level of oxygen vacancies before the reduction. Nevertheless the crucial observation is that, after the back-substitution, the superconducting transition temperature of the back-substituted samples has become nearly the same. This allows us to conclude that the *T*_*c*_ in SrTiO_3_ is tuned by the mass of oxygen isotope.

### Isotope effect on *T*_*c*_, *H*_*c*2_, *υ*_*F*_ and *λ* due to polaronic band-narrowing

Due to the anti-adiabatic conditions, the expression for *T*_*c*_ in the relevant doping range reads^9^


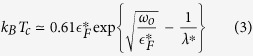


with 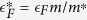 the Fermi temperature of the polaron Fermi-liquid. Since 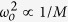, the oxygen isotope coefficient for *T*_*c*_ is


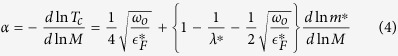


The upper critical field in the clean limit is


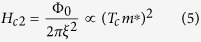


from which we readily obtain for the isotope coefficient of the upper critical field


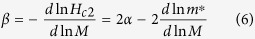


Another parameter showing isotope effect is the Fermi-velocity, for which


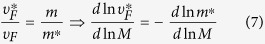


This can in principle be measured with high resolution angular resolved photo-emission. The isotope effect of the London penetration depth is described by


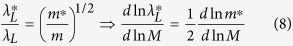


which can in principle be measured using *μ*SR.

For SrTiO_3_ the polaron mass renormalization m*/*m* is of intermediate strength^7^ for which 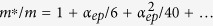, where *α*_*ep*_ is the electron-phonon coupling strength causing the mass-renormalization and the expansion presupposes weak coupling (*i.e. α*_*ep*_ < 6)^45^. We combine this with the property of the electron-phonon coupling constant 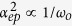, to describe the oxygen-isotope dependence of the polaron-mass as a function of m*


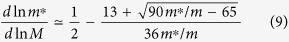


## Additional Information

**How to cite this article**: Stucky, A. *et al*. Isotope effect in superconducting n-doped SrTiO_3_. *Sci. Rep.* **6**, 37582; doi: 10.1038/srep37582 (2016).

**Publisher's note:** Springer Nature remains neutral with regard to jurisdictional claims in published maps and institutional affiliations.

## Figures and Tables

**Figure 2:**
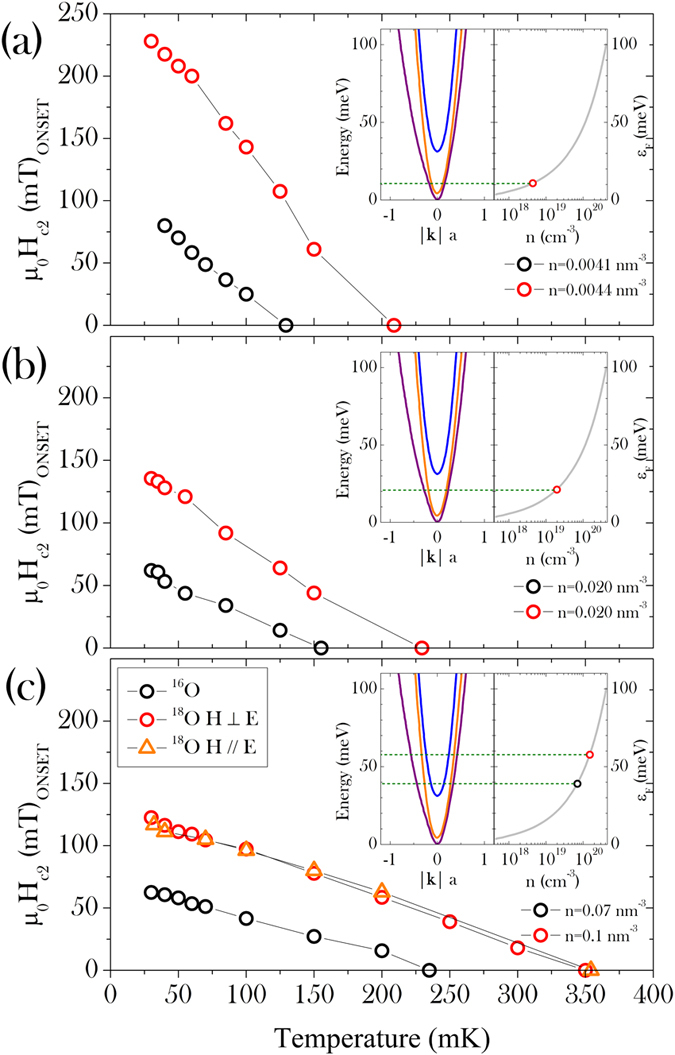
Upper critical field as a function of temperature at various doping levels. Black symbols: SrTi^16^O_3−*y*_; red symbols: ^18^O-substituted SrTiO_3−*y*_. Panels (a–c) refer to charge carrier densities *n* ~ 0.004, 0.02, 0.07 nm^−3^, respectively. Open orange triangles and red circles in panel (c) indicate the H_*c*2_ values measured with two mutually perpendicular directions of the applied magnetic field, both being perpendicular to the flowing current. Insets: detail of the conduction bands of SrTiO_3_ and Fermi energy at each doping level^9^.

**Table 1:** Parameters for doped SrTiO_3_.

n nm^−3^	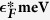	λ*	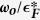	*d* ln m*/*d* ln *M*	*α*	*β*
0.004	2.9	0.091	17	0.25	−2.0	−4.5
0.020	7.1	0.104	7	0.24	−1.8	−4.1
0.100	20	0.115	2.5	0.20	−1.3	−3.0

For the fourth column *ħω*_0_ = 50 meV was used. See ref. 9 for the values of *ϵ*_*F*_ as a function of carrier concentration. The the log-derivatives of the mass and the *α*- and *β*-coeffients and were calculated with Eqs 9, 4 and 6 (Methods) respectively.

**Table 2:** Sample identifier, oxygen isotope, annealing temperature, annealing time, charge carrier density measured from the Hall constant and residual resistance ratio (RRR = R(300 K)/(R(4 K)) of the samples reported in this manuscript.

Sample	oxygen isotope	Temp *°C*	time *hours*	n *nm*^−3^	RRR
MTI-1	^16^O_2_	1050	36	0.0041	1383
MTI-2	^16^O_2_	1200	36	0.020	389
MTI-3	^16^O_2_	1350	36	0.070	106
MTI-4	^18^O_2_	1050	36	0.0044	1000
MTI-5	^18^O_2_	1200	36	0.012	388
MTI-6	^18^O_2_	1350	36	0.15	45

**Table 3:** Isotope content in various samples as obtained from mass spectroscopy.

Samples	% of ^16^*O*	% of ^18^*O*
MTI-0 ^16^*O*	99.79	0.21
MTI-0 ^18^*O*	91.81	8.19
MTI-1	99.72	0.28
MTI-4	61.18	38.82
MTI-2	99.68	0.32
MTI-5	65.21	34.79
ceramic ^16^*O*	99.35	0.65
ceramic ^18^*O*	65.90	34.10
